# *Viewing the Invisible*: Exploring common methodology across disciplines

**DOI:** 10.1371/journal.pbio.3000577

**Published:** 2019-12-16

**Authors:** Ann Witheridge, Rivka Leah Isaacson

**Affiliations:** 1 London Fine Art Studios, London, United Kingdom; 2 Department of Chemistry, King’s College London, London, United Kingdom

## Abstract

*Viewing the Invisible* is a multimedia collaboration that explores common methodology between arts and sciences. Portraits of science influencers were painted while dialogue between artist and subject was filmed. Here, we show the implementation of our recent exhibition as a model that can be adapted for use elsewhere in public or school settings to challenge misconceptions about the role of creativity in science and technical precision in art.

## Abstract 2

This Community Page article describes “Viewing the Invisible,” a multimedia collaboration where artists painted portraits of scientists, mutually discovering the creativity of science and the technical precision of art.

## Background

The United Kingdom education system is prone to bifurcating students along either a science or humanities path from an early age, sometimes as young as 13 [1]. If sufficiently motivated, it is possible to rediversify at most stages, but on the whole, these early choices influence the course of entire careers, and anecdotally, it is rare to find people equally open-minded about science and arts. Although the relationship between the two fields has fluctuated over time, they are often considered to reside at polar ends of a spectrum [2]. However, there is a renewed interest in considering their overlap and interplay in areas such as visual representation, creativity, and methodology [3].

## Project and goals

**Fig 1:**
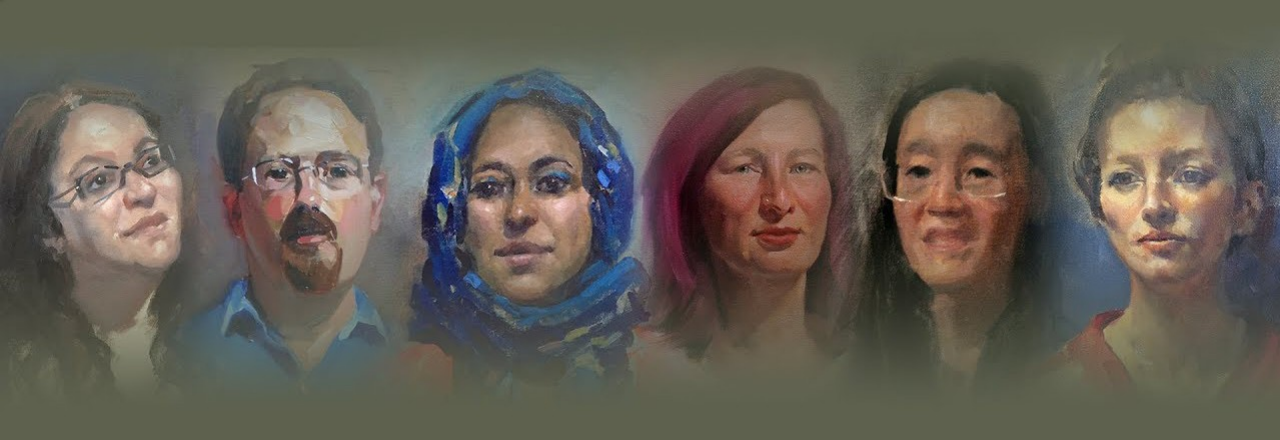
*Viewing the Invisible* project banner featuring portraits left to right of Naomi Alderman, Julian Huppert, Misbah Arif, Rivka Isaacson, Chun-wa Chung, and Megan Dowie. (*Image*: *acapmedia*).

*Viewing the Invisible* is a project designed to explore common ways of working between scientists and artists. We chose six “science influencers” from different walks of life (education, politics, media, industry, academia, policy) to have their portraits painted by artists from London Fine Art Studios (Fig 1). We made short films of the sessions, which incorporated discussions between painter and sitter about their respective practice and preconceptions. To minimize costs, we took stills and silent movies during the painting process and then filmed each pair in conversation for about 20 minutes, during which they revisited issues around “ways of working” that arose from their earlier conversations. In collaboration with the videographers, acapmedia, we then edited all the best parts the into final films. Our goals are to highlight common thinking, practice, and motivation in science and art and to start conversations about this through our exhibitions and online films.

## Observations

Perhaps because of the intimate nature of the scenario, a remarkable rapport built up between each artist/sitter combination, as evidenced in the short films. Extensive common ground was identified, and everyone involved in the project reported having enjoyed the mind-opening experience and repairing some former misconceptions.

Unusually, and somewhat by design, many of our participants already straddle the science and art worlds, including painter Clementine St. John Webster, whose first degree was in biology. She painted Megan Dowie, who has a PhD in neuroscience, has a longstanding interest in art and outreach and now works in science funding. Megan observed, “We both use both disciplines in our professional roles and the different creative ways of thinking influence how we work now.” Naomi Alderman was formally educated in humanities and is a novelist, alongside tech writing and presenting science radio. Science teacher Misbah Arif paints as a hobby and experiences first-hand the sectarian attitudes to arts and sciences exhibited by the UK education system [4].

**Fig 2:**
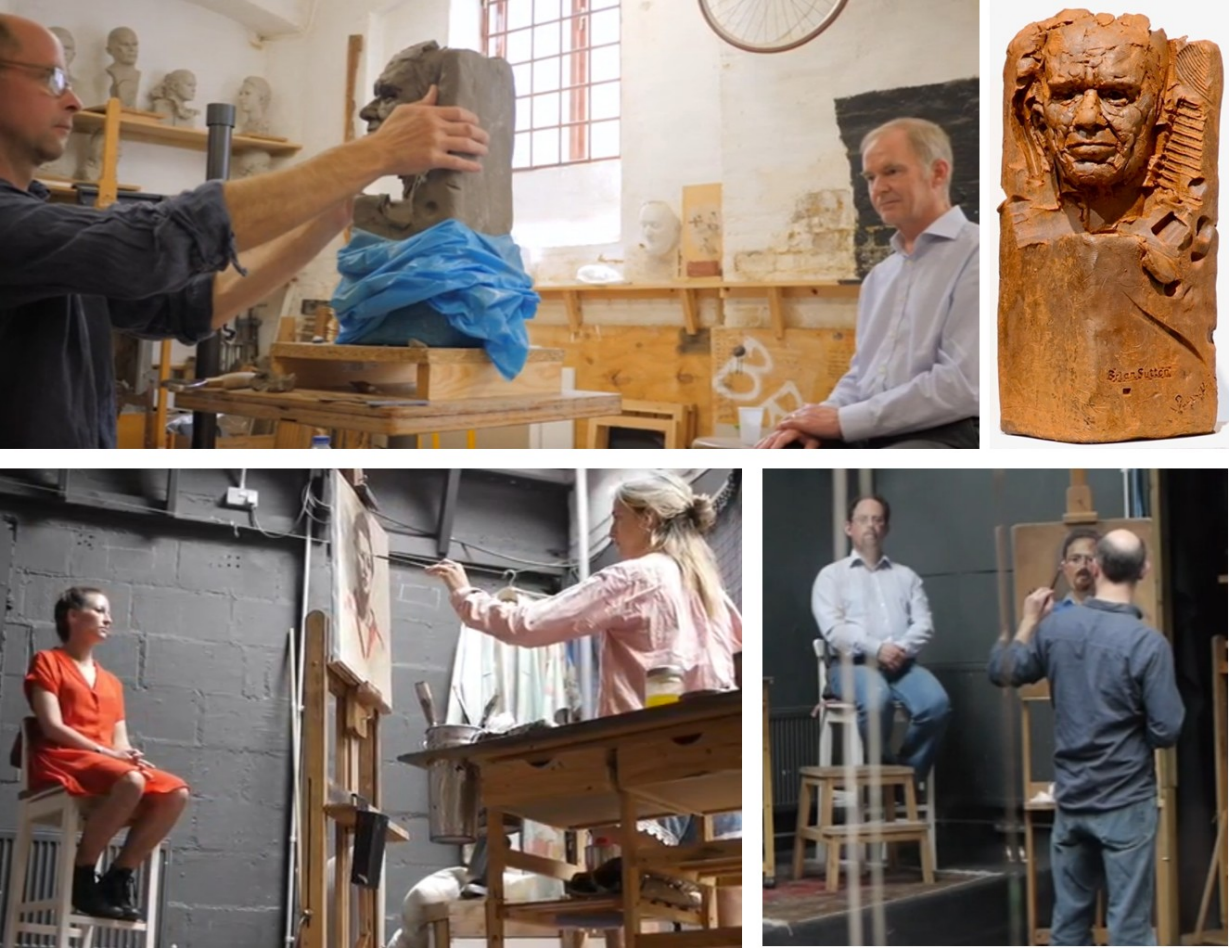
(Top left) “It was an extraordinary experience being looked at by you.” Brian Sutton sculpted by Scott Pohlschmidt. (Top right) Finished sculpture cast in resin. (Bottom left) Clemmie St. John Webster viewing/painting Megan Dowie. (Bottom right) Scott viewing/painting Julian Huppert.

Several common threads emerge from the discussions. Both disciplines face related challenges of representing three-dimensional objects in two dimensions. Another theme is talent, brainpower, and perseverance—how far can people go in either science or art, if they set their minds to it? Several sitters commented on the novel experience of being “looked at” so intently to facilitate the process of depiction rather than for human interaction (Fig 2). Both artists and scientists use unrealistic colors to convey entirely natural objects. Scientists were surprised by the technical precision required for portrait painting and artists by the myriad avenues for creativity open to the scientists in their work. This is exemplified in many quotes from the films, including artist Clemmie, “I love the thought that light stimulus lands on our retina, converts into electrical activity that whizzes round our brain, converts a conscious experience into an actual physical painting which then tries to evoke a similar experience in the viewer”; scientist Chun-wa Chung, “You are more technically passionate than I would ever have expected”; and artist Joni Duarte, “Understanding how light works—it’s all about physics. If it’s a pigment problem, it’s chemistry.”

## Exhibition

**Fig 3:**
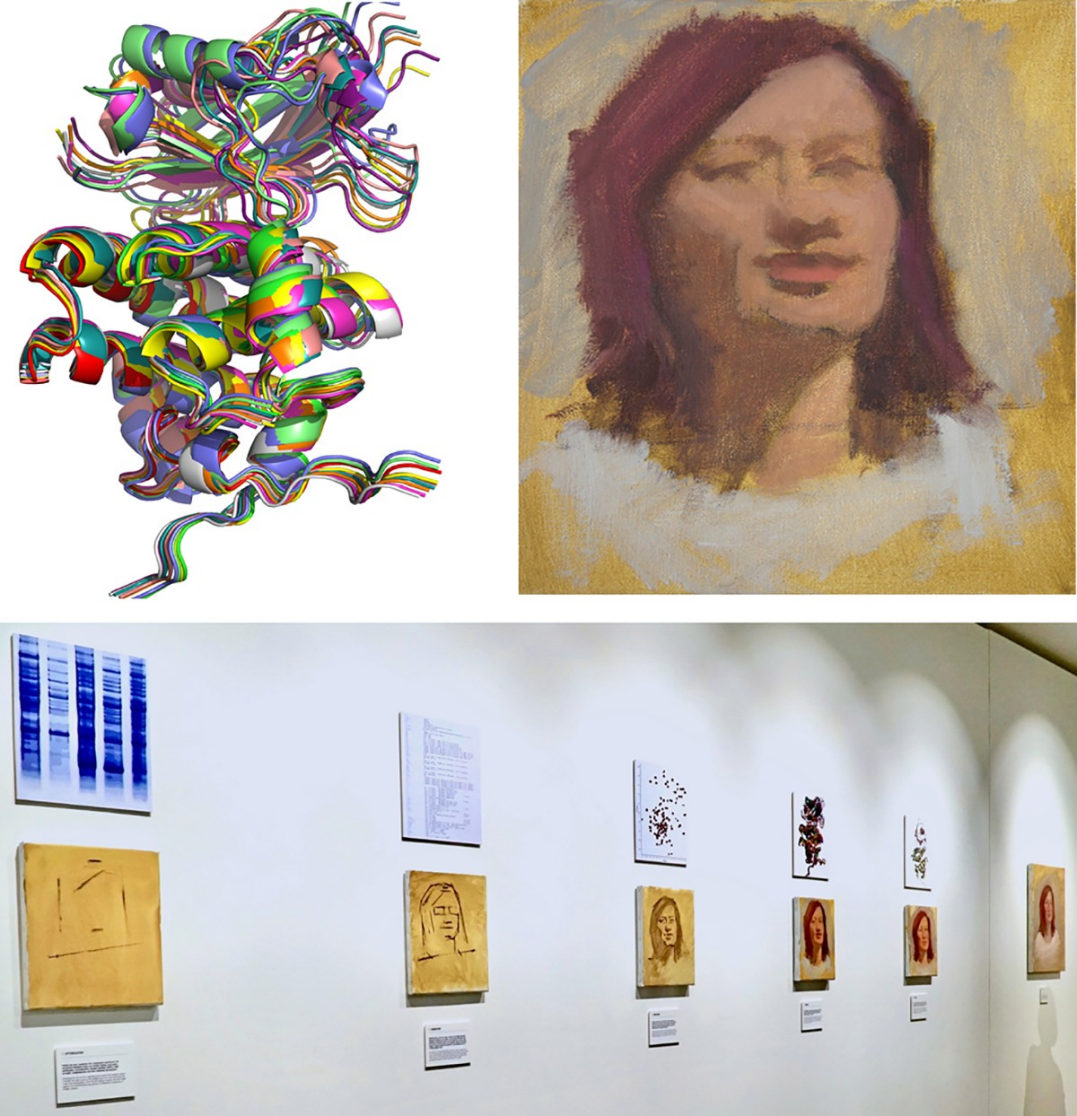
(Top) A sample storyboard image in which creative stages were compared. This step illustrates “Iteration.” (Left) An ensemble of protein structures with caption: “Each calculation we run, teaches us something more about the things we have got right and the things we have got wrong. Going back to implement each newfound set of knowledge brings us closer to the truth in iterative cycles of validation.” (Right) The first stage in portraiture in which color is introduced with caption, “Lay in the big areas of colour, one for the lights, the dark, the hair and the background. By keeping the colours simple we create a harmony that runs throughout the painting. Once we are happy find a darker value in your darks and a lighter value in your lights, pushing the value pattern and form a little more.” (Bottom) The storyboard wall in situ (*Photo*: *Nada Walton*).

We created a free exhibition that went on display in a public art gallery, The Arcade, Bush House on the Strand in London, in September 2019, a period culminating in “welcome week,” the first week of term for new undergraduates. The exhibition comprised the six portraits, short films, the sculpture, and a series of storyboarding canvases tracing parallels between stages of the creative process in science and art. These ranged from choosing a subject; convincing others of its importance; generating resources; finding optimal conditions; data-acquisition, processing, iteration (Fig 3), and refinement; and finally, dissemination. We gathered data from visitors via a questionnaire, which included questions about their favorite exhibit, whether/how their views had been changed or enhanced, and whether/how they might now adjust their personal or professional practice.

A detailed analysis of the responses is beyond the scope of this short article, but every aspect of the exhibition turned out to be at least someone’s top pick, and many respondents reported opening their minds as a result of our show. Several scientists voiced plans to take up or revisit artistic practices, with guests from the humanities side wanting to learn more about science and develop collaborations with scientists. Teachers said they will encourage their students to think about different fields in a more integrated way.

## Events

We held an accompanying 90-minute event at the National Portrait Gallery in Trafalgar Square in which artist Ann Witheridge live-painted a portrait of Professor Dame Janet Thornton FRS, a founder of the field of bioinformatics. The event included a panel discussion on science and art by several participants from *Viewing the Invisible* and formed part of the gallery’s public programming on the theme of identity, in parallel with their iconic Cindy Sherman exhibition. During welcome week, we held workshops for new students, drawing and painting scientific subjects.

## Practical considerations for duplication

We would be delighted if others chose to run similar projects in their own environments. It can be scaled up or down depending on budget. In this respect, we had much greater success adding UK£10,000 “impact” budgets to standard science project grant applications than by laboring over specific “public engagement” grant schemes. Once funded, the biggest challenge is to identify portrait artists who can work at speed, achieving a credible likeness, as it is hard to convince busy people to sit for any length of time. We would recommend researching classical portraiture schools in your nearest cities or contacting us for help. Over the years, London Fine Art Studios has trained many artists, who are now scattered all over the world, so we can make connections. If films are required, which we highly recommend, then videographers must also be identified. The videos have hugely expanded the reach of our project; they help us pitch the exhibition to venues and form a lasting legacy, which will hopefully now reach a wider international audience as a result of this paper. Approach some local science heroes in different spheres of influence and ask if they are willing to be painted in exchange for the final gift of their portrait once any exhibitions have concluded. We have learned that if the request is framed with sufficient sheepishness, even the most reluctant subject can be persuaded to sit for a portrait. Our largest costs were the videographers, staffing the exhibition (not strictly necessary but highly effective in engaging passersby and persuading visitors to complete the feedback survey, the results of which are usually necessary to demonstrate impact), and refreshments/staff for the private view. We also paid for art materials and artists’ fees for painting the portraits and delivering workshops.

## Future plans

In the future, we will take *Viewing the Invisible* on tour and exhibit it in other UK cities or farther afield. We also plan to adapt it into a social action plan to improve uptake of science education, particularly by young girls, in underprivileged educational settings. We will bring a female scientist into a school and run a live-painting session in which an artist will paint the scientist’s portrait amid discussion between artist and sitter about their respective methodology.
